# Severe Initial Presentation of Systemic Lupus Erythematosus Mimicking Hemophagocytic Lymphohistiocytosis Secondary to Epstein-Barr Virus (EBV): A Case Report

**DOI:** 10.7759/cureus.100085

**Published:** 2025-12-25

**Authors:** James Di Palma-Grisi, Alexander Vallone, Sophia Zhang, Egor Volcotrub, Danit Arad

**Affiliations:** 1 Internal Medicine, Hackensack University Medical Center, Hackensack, USA; 2 Internal Medicine, Hackensack Meridian School of Medicine, Nutley, USA

**Keywords:** epstein barr virus, hemophagocytic lymphohistiocytosis, hoagland's sign, subacute appendicitis, systemic lupus erythematosus

## Abstract

A Hispanic woman in her fifth decade of life presented to the Emergency Department with severe abdominal pain, dry heaving, fevers, and weight loss for two weeks. She underwent a CT of the abdomen and pelvis, notable for appendiceal thickening, and was diagnosed with subacute appendicitis with regional lymphadenopathy, thought to be reactive. Her fever persisted on empiric antibiotics, and she underwent a total-body CT that showed diffuse lymphadenopathy. She developed pancytopenia, underwent a bone marrow biopsy, and was started on high-dose dexamethasone with concern for hemophagocytic lymphohistiocytosis (HLH). Her symptoms improved on dexamethasone but remained persistent throughout her tapering, and she underwent rheumatological evaluation given her family history of systemic lupus erythematosus (SLE) in her adult daughter. She was found to have positive anti-double-stranded DNA (anti-dsDNA) antibodies, was started on hydroxychloroquine, improved, and was discharged from the hospital with outpatient rheumatology follow-up. Later, her kidney biopsy was positive for lupus nephritis, confirming the diagnosis.

## Introduction

Acute appendicitis is typically due to obstruction of the appendiceal lumen and subsequent inflammation of the vermiform appendix. It often manifests with fever, periumbilical pain that migrates to the right lower quadrant, nausea, and vomiting. The gold-standard imaging is a CT of the abdomen and pelvis, and surgical consultation is required for safe assessment [1].

Although rare, subacute appendicitis can be caused by an Epstein-Barr Virus (EBV) infection, which is reported to have infected more than 90% of adults worldwide. EBV classically presents as infectious mononucleosis, with fever, pharyngitis, and cervical lymphadenopathy for a period of two to four weeks’ time [2]. Other symptoms can include splenomegaly, diffuse abdominal pain, hepatomegaly, and a maculopapular rash. EBV serology confirms the diagnosis, and although clinical trials are ongoing for specific antiviral treatments, the current standard of care is conservative management in an immunocompetent host [3]. EBV is an oncovirus that can cause lifelong, persistent infection and can transform and immortalize B cells, driving malignancies including Burkitt lymphoma, Hodgkin lymphoma, and nasopharyngeal carcinoma [4]. Both EBV and its associated lymphomas can also be associated with hemophagocytic lymphohistiocytosis (HLH) and autoimmune hemolytic anemia [5].

HLH is primarily broken into two major subgroups, with distinct pathophysiology. Primary HLH is congenital and caused by cellular miscommunication between unprimed CD8+ cytotoxic T cells and the various antigen-presenting cells (APCs) of the human body. Because of this dysregulation, T cells are ineffective in removing antigens that trigger an immune response, which results in runaway production of proinflammatory cytokines, as APCs continue to try and recruit effector T cells. One result of this dysfunction is the systemic activation of macrophages, leading to broad cellular destruction, and this severe presentation is known as macrophage activation syndrome (MAS). Secondary HLH is an immune dysregulation that is triggered by an acute illness rather than a genetic mutation. Normally, patients with secondary HLH are over 50 years old and include secondary infections such as tuberculosis, fungal infections, and histoplasmosis, as well as malignancies such as T-cell lymphomas and B-cell lymphomas [6]. 

Systemic lupus erythematosus (SLE) is an autoimmune disease that commonly affects multiple organ systems via dysregulation of both the innate and adaptive immune responses. This results in persistent exposure of nuclear antigens, which become identified as autoantigens and promote autoantibody production, classically anti-double-stranded DNA (anti-dsDNA) and anti-nucleoprotein antibodies (ANAs). SLE can manifest with rashes, including the classic malar rash over both cheeks and the bridge of the nose, fatigue, fever, loss of appetite, abdominal pain, myalgias, arthralgias, and nephritic syndrome [7]. Patients with SLE have a higher prevalence of active EBV infection, higher EBV viral loads, and increased seropositivity for EBV antigens compared to controls, suggesting that EBV may be an independent risk factor for later development of SLE [8]. Given the vast array of symptoms that SLE can produce, and its overlap with other rheumatological and non-rheumatological diagnoses, delayed diagnosis and treatment are unfortunately common occurrences. The average delay from initial presentation to diagnosis was 47 months in one study - longer delays were associated with disease activity, end-organ damage, fatigue, and impaired quality of life [9].

## Case presentation

A Hispanic woman in her fifth decade of life presented to the Emergency Department (ED) with diffuse abdominal pain, dry heaving, intermittent fevers, and weight loss for two weeks. She said her symptoms came on all at once and that she had never had them before. She had been eating less due to the dry heaving and attributed the weight loss to this. She initially took ibuprofen at home without relief and went to her primary care provider (PCP), who advised her to have an ultrasound of her abdomen done. She then felt constipated for the past few days, had one episode of non-bloody diarrhea, and presented to the ED because her symptoms were not improving, at the prompting of her daughter, with whom she lives. She denied headache, rhinorrhea, cough, shortness of breath, chest pain, dysuria, leg swelling, unwell contacts, recent travel, or any changes to her diet. She works in a facility that processes food but does not handle any food products herself. She had never been incarcerated or unhoused. Her past medical history was notable for hypothyroidism and restless leg syndrome, and her only home medication was levothyroxine 25 mcg once daily. Her family history was notable for SLE in her adult daughter.

**Figure 1:**
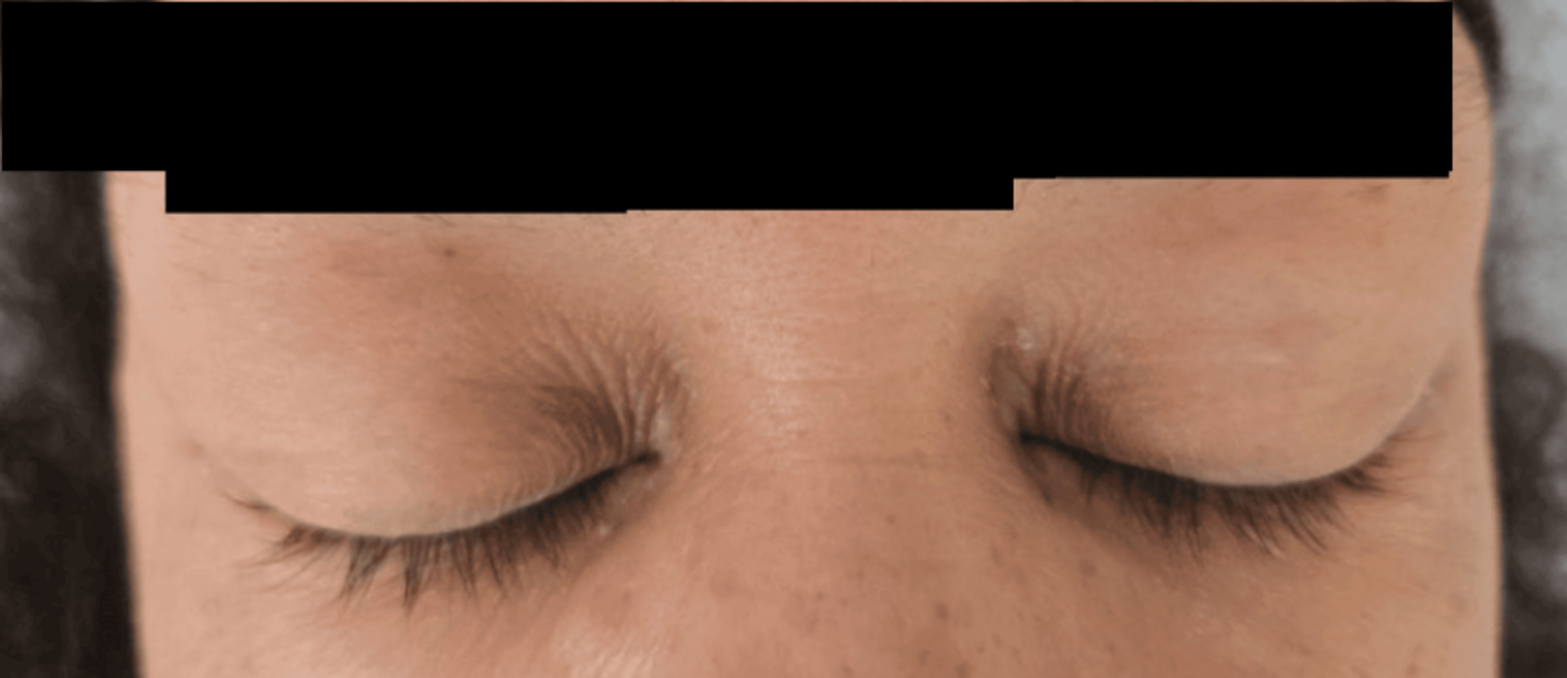
Hoagland sign: bilateral upper eyelid swelling in a characteristic S-shape, looping around the outer eyelids, closely associated with infectious mononucleosis.

Her vital signs upon ED presentation were a temperature of 39.4 °C, pulse of 146, respiratory rate of 34, blood pressure of 108/55, and oxygen saturation of 95%. She continued to dry heave, vomit, and complain of severe abdominal pain predominantly over her right lower abdomen. On physical exam, she had bilateral upper eyelid swelling (Figure 1), tachycardia, normal S1 and S2, no murmurs, rubs, or gallops, clear lung sounds bilaterally, no wheezes, rales, or rhonchi, diffuse abdominal tenderness over all four quadrants, voluntary guarding, no rebound tenderness, no costovertebral angle tenderness, no pretibial, ankle, or pedal edema, capillary refill of less than two seconds, normal-appearing skin without jaundice or pallor, and she was alert and oriented to person, place, time, and hospital course.

**Figure 2:**
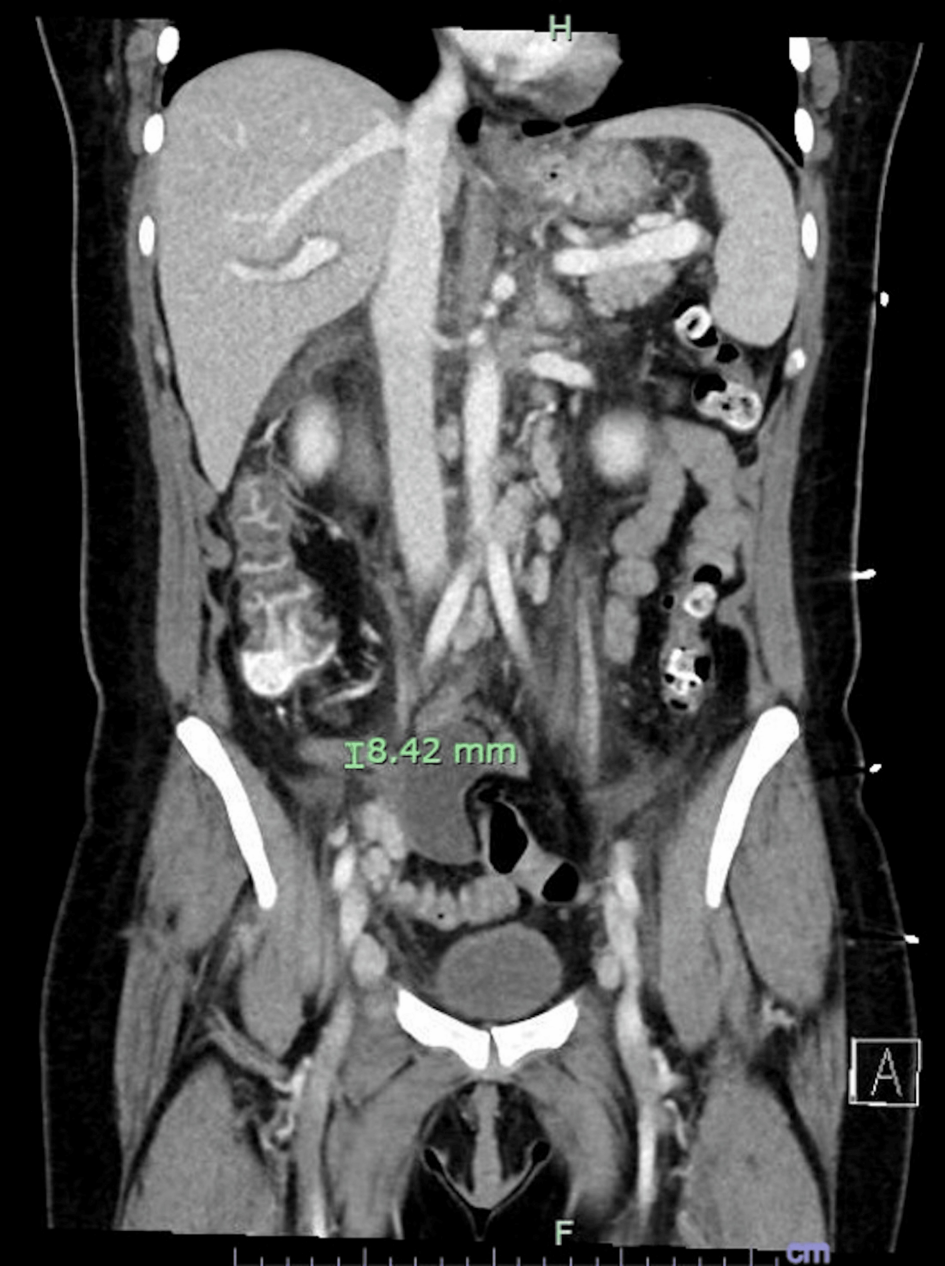
CT of the abdomen and pelvis with contrast, showing appendiceal thickening (marked, 8.42 mm) and diffuse lymphadenopathy.

Complete blood count with differential indicated pancytopenia. EBV antibody titers were positive for IgM and IgG, favoring a late stage of acute infection. Human immunodeficiency virus (HIV), cytomegalovirus (CMV), parvovirus B19, and hepatitis virus panels were negative. Flow cytometry was negative, and the lymph node core biopsy was also normal. Blood smear was normal. CT of the abdomen and pelvis indicated a thickened appendix and abdominal, retroperitoneal, and pelvic lymphadenopathy (Figure 2). Our differentials were initially broad, as the patient presented with nausea, vomiting, diarrhea, and severe, acute-on-chronic abdominal pain. Her initial CT of the abdomen and pelvis supported a diagnosis of appendicitis, and the abdominal lymphadenopathy was thought to be secondary to her appendiceal inflammation. Surgical consultation agreed with the diagnosis and advised medical management, observation, and antipyretics. She was treated with three days of ceftriaxone 2 g IV once daily and metronidazole 500 mg IV three times a day. Her symptoms initially improved, then worsened after stopping these antibiotics. She continued to have fever and experience abdominal pain and was treated with a further three days of levofloxacin 750 mg IV once daily and metronidazole 500 mg IV three times a day. 

After several days of IV antibiotic treatment, she remained nauseous and continued to have fever. While she was undergoing treatment, her EBV polymerase chain reaction (PCR) was positive, and EBV serology reported positive IgM and IgG, favoring a late stage of infection. Flow cytometry was negative for circulating blood cell malignancies. Infectious diseases consultation agreed with active EBV infection but noted that, while it may produce the radiographic findings of appendiceal thickening and diffuse lymphadenopathy, given her presenting history, an acute infection should have symptomatically resolved with supportive care. Around one week into her hospital course, she developed worsening pancytopenia. In view of her EBV infection, constitutional symptoms, and nonresolving fevers, we became concerned that this was a first presentation of an aggressive lymphoma. Her subacute presentation, with predominant night sweats, nonspecific abdominal pain, and intermittent non-infectious fevers, supported this diagnosis.

We ordered a lymph node biopsy under CT guidance and peripheral flow cytometry to further characterize this risk. We also sent a diagnostic blood test panel to calculate an H-score to assess the probability of HLH, which was above the cutoff of 169 and suggestive of HLH (Table 1). Specifically, the fever, hepatomegaly, pancytopenia, high ferritin, high triglycerides, and high aspartate aminotransferase (AST) resulted in a score of 203, conferring an 88%-93% likelihood of HLH. Although ferritin and triglycerides are acute-phase reactants, ferritin levels in the tens of thousands are closely associated with autoimmune diseases and hematologic malignancies. The hyperinflammatory and hyperfibrinolytic state associated with HLH can also drive diffuse intravascular coagulation (DIC) and associated low fibrinogen levels.

**Table 1:** Admission blood and urine tests with results; HLH panel testing on day 7 of admission. HLH, Hemophagocytic lymphohistiocytosis; ALP, Alkaline phosphatase; ALT, Alanine aminotransferase; AST, Aspartate aminotransferase

Initial Labs (Day 1)		HLH Labs (Day 7)	
White Cell Count	2.8 x 10^9^/L	White Cell Count	3.2 x 10^9^/L
Hemoglobin	109 g/L	Hemoglobin	86 g/L
Platelet Count	70 x 10^9^/L	Platelet Count	36 x 10^9^/L
Sodium	133 mmol/L	Ferritin	>33,511 ng/mL
Potassium	3.6 mmol/L	Fibrinogen	194 mg/dL
Chloride	98 mmol/L	Triglycerides	307 mg/dL
Bicarbonate	26 mmol/L	AST	346 U/L
Urea	8 mg/dL	Soluble IL-2 (CD25)	5,248 pg/mL
Creatinine	0.75 mg/dL	Urinalysis (Day 1)	
Calcium	8.4 mg/dL	pH	6.5
Magnesium	1.53 mg/dL	Specific Gravity	1.020
Phosphorus	2.3 mg/dL	Glucose	Negative
Albumin	3.6 g/dL	Blood	Negative
Lipase	3.2 U/L	Ketones	80 mg/dL
ALP	107 U/L	Protein	100 mg/dL
AST	110 U/L	Bilirubin	Negative
ALT	41 U/L	Nitrites	Negative
Lactic Acid	1.1 mmol/L	Leukocyte Esterase	Negative
Ferritin	9784 ng/mL	White Blood Cell	3-5/HPF
Fibrinogen	274 mg/dL	Red Blood Cell	0-2/HPF
Triglycerides	124 mg/dL	Bacteria	Few
Blood Cultures	Negative at 5 days	Squamous Cells	Few

We consulted the hematology service, which agreed the patient met the diagnostic criteria for HLH and performed a bone marrow biopsy, but advised treatment with steroids alone, as it was felt to be secondary to an underlying condition and would not be likely to respond to etoposide treatment (as primary HLH would). She was treated with five days of dexamethasone 20 mg PO twice a day, with improvement in her symptoms. She was unable to wean to oral prednisone, however, and was instead transitioned to methylprednisolone 32 mg PO daily, with continued symptom improvement. 

**Figure 3:**
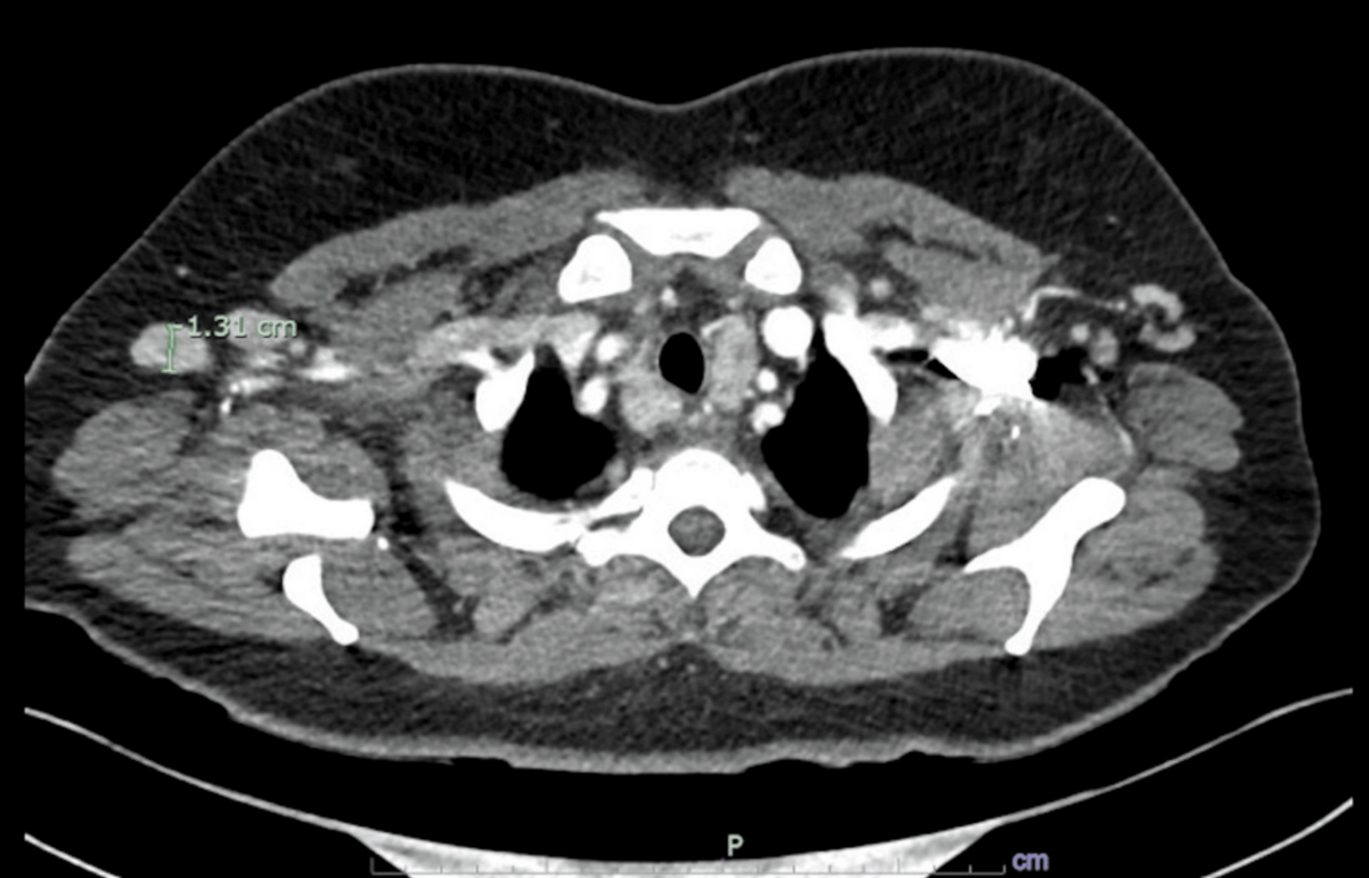
CT chest with contrast showing enlarged bilateral axillary lymph nodes measuring up to 1.31 cm in the short axis on the right (marked), as well as an increased number of subcentimeter mediastinal lymph nodes without pathologic enlargement.

Around one month into her hospital course, she developed a facial rash in a classical malar pattern. This reopened our differential to other causes of noninfectious fever. CT of the neck and chest were notable for axillary and mediastinal lymphadenopathy, and a lymph node core biopsy was taken from her right axillary lymph node (Figure 3). Her initially mild transaminitis worsened, and a right upper quadrant ultrasound showed mild gallbladder thickening and trace pericholecystic fluid, thought to be secondary to EBV viremia, on general surgical consultation.

We explored the possibility that she could have a late initial presentation of SLE in view of her family history and took a disease-specific history that disclosed chronic hair loss, initially attributed to iron deficiency anemia. After thoroughly investigating the possibility of leukemia, lymphoma, and HLH, we consulted rheumatology and sent anti-Sjögren's-syndrome-related antigen A autoantibodies (anti-SSA), anti-Sjögren's-syndrome-related antigen B autoantibodies (anti-SSB), anti-dsDNA, C3, and C4 markers, all of which were suggestive of an active SLE flare.

Her ANA was positive 1:80 with a cytoplasmic, fibrillar pattern, and 1:1280 with a nuclear, speckled pattern; her anti-dsDNA was positive 1:40, her C3 was 8 mg/dL, and her C4 was 3 mg/dL. dsDNA was equivocal, disfavoring an active SLE flare, but C3 and C4 were reduced, favoring an active flare. She was continued on methylprednisolone and started on hydroxychloroquine 200 mg PO daily. Kidney biopsy was notable for diffuse mesangial proliferative glomerulonephritis, confirming the diagnosis of lupus nephritis.

Her inpatient course rapidly improved after her initial five-day course of high-dose dexamethasone, as her nausea, fevers, and abdominal pain resolved. Her transaminitis also improved on her maintenance methylprednisolone and hydroxychloroquine. Her alkaline phosphatase (ALP), AST, and alanine aminotransferase (ALT) peaked at 216 U/L, 250 U/L, and 99 U/L, and dropped to 130 U/L, 41 U/L, and 42 U/L on discharge, respectively. As she remained on high-dose steroids, famotidine 20 mg PO twice a day and sulfamethoxazole-trimethoprim 800-160 mg PO three times a week were added upon discharge. She continued to experience diarrhea and arthralgias after discharge, and she was started on mycophenolate mofetil, with improvement in these symptoms as well. She returned to work approximately two weeks after discharge and follows up every three months with her rheumatologist.

## Discussion

This patient was admitted with symptoms most consistent with appendicitis and underwent a complex clinical course that revealed systemic autoimmune pathology. We would divide the differential diagnosis into three distinct periods: her initial presentation with predominantly gastrointestinal symptoms and severe abdominal pain, her persistent fevers with diffuse lymphadenopathy unresponsive to antibiotics, and her pancytopenia with a normal bone marrow biopsy (Table 2).

**Table 2:** Evidence supporting the principal differential diagnoses. In the first week of admission, we considered subacute appendicitis vs. infectious mononucleosis. When she developed worsening pancytopenia and fevers unresponsive to antibiotics, we considered HLH, and later, SLE. HLH, Hemophagocytic lymphohistiocytosis; SLE, Systemic lupus erythematosus; Anti-dsDNA, Anti-double-stranded DNA

	Clinical Features	Lab Testing	Imaging/Pathology
Appendicitis	Abdominal pain, nausea, vomiting	Leukocytosis	Appendiceal thickening
Epstein-Barr Virus (EBV), Burkitt Lymphoma	Night sweats, fevers, unintended weight loss, Hoagland sign	Positive anti-EBV IgG, IgM	Lymphadenopathy beyond what would be expected in appendicitis (later cervical/thoracic)
HLH	Altered mental status, malaise	Anti-CD25, high ferritin, low fibrinogen and triglycerides	Hepatomegaly
SLE	Malar rash, hair loss	Anti-dsDNA, reduced C3 and C4 levels	Kidney biopsy positive for lupus nephritis

In terms of her immediate presentation, there was a meta-analysis of medical vs. surgical management of uncomplicated appendicitis that found medical management with antibiotics was associated with shorter recovery time, fewer complications, and less pain, but that the overall efficacy was lower in view of the high rate of recurrence, with a mean overall recurrence rate of 13% [10]. In view of her subacute presentation and diagnostic uncertainty, we feel the initial surgical consultation and choice to proceed with medical management, after risk stratification, was reasonable. Her initial improvement with antibiotics supported this diagnosis, pending further workup of her diffuse lymphadenopathy [11].

The next phase of care revolved around the workup and treatment of EBV-HLH. She met the diagnostic criteria for HLH, and her altered mental status, malaise, highly specific findings, such as anti-CD25 levels, and known trigger of acute EBV infection all supported this diagnosis. However, one inconsistency was the time course of her symptoms - if she had secondary HLH, she would have likely appeared far more unwell much earlier in her clinical course, even before the bone marrow biopsy results were available. Likewise, her subacute presentation, rash, alopecia, and anti-dsDNA levels, combined with her intermittent clinical course responsive to steroids, favored a chronic, autoimmune etiology more typical of SLE. 

Our clinical suspicion for active EBV was primarily driven by her positive antibody titers, her subacute, nonspecific presentation with B symptoms, and her positive Hoagland sign. EBV can cause eyelid problems, most notably the Hoagland sign, which is painless, bilateral upper eyelid swelling that causes drooping of the outer part of the eyelid, creating an “S-sign.” This may have been her true initial presentation, but EBV is known to drive active SLE flares through a complex mechanism, including activation of autoreactive T cells and epitope spreading among naive B cells [12]. The initial concern for HLH was based on her worsening pancytopenia, and her initial response to high-dose steroids convinced the treatment team and consulting hematology service that the most appropriate diagnosis was EBV-driven HLH without associated malignancy. Upon further clinical review, the hematology service actually noted the malar rash that had developed in the course of her hospitalization, prompting additional testing specifically for SLE.

Given her family history of SLE, a late initial presentation could have been suspected earlier in her clinical course, and there are individual case studies reporting pancytopenia due to active SLE flares [13]. Similarly, the mortality rate associated with HLH secondary to underlying malignancies is approximately 80%, making early recognition and treatment critical [14]. SLE can be associated with other autoimmune-mediated bone marrow dysfunction, most notably antiphospholipid syndrome, which can be associated with bone marrow necrosis [15]. SLE can also be associated with anemia of chronic disease. One review of hematological associations with SLE found that 13% of patients developed acute hemolytic anemia, 20% developed lymphopenia, and 27% developed thrombocytopenia; MAS, a severe form of secondary HLH, has also been reported in patients with SLE [16]. Overall, the most recent diagnostic criteria for HLH and SLE can have significant overlap. For example, leukopenia, thrombocytopenia, and fever award 9 of 10 points needed for a diagnosis of SLE, along with positive ANA titers [17]. The same two-cell-line cytopenia and fever would award 2 of the 5 positive criteria needed to diagnose HLH based on the revised HLH-2024 criteria [18].

Some limitations in our diagnosis include the influence of active EBV infection on autoimmune markers, for example, or the possibility of SLE and mixed connective tissue disease (MCTD), which could also confound the diagnosis and include positive autoantibodies like anti-dsDNA [19]. We feel that, due to the considerable overlap between the common presentations of both conditions, testing for one condition often warrants testing for the other in the absence of known triggers for HLH (e.g., malignancy, infections like EBV, immunosuppression). In hindsight, the initial EBV infection, as evidenced by positive PCR and IgM titers, may have triggered her severe SLE flare. Identification of positive anti-dsDNA antibodies and biopsy-proven lupus nephritis confirmed the diagnosis, emphasizing the importance of thorough autoimmune workup when clinical suspicion persists, despite inconclusive initial findings. 

## Conclusions

SLE can present with atypical and nonspecific symptoms, such as fever, abdominal pain, and weight loss, which may mimic surgical emergencies like appendicitis. Persistent fever and diffuse lymphadenopathy unresponsive to antibiotics should prompt evaluation for autoimmune, malignant, or hematologic etiologies, as in our case. Pancytopenia in the setting of systemic inflammation should raise concern for secondary HLH, a life-threatening condition that may be triggered by underlying autoimmune disease. A multidisciplinary approach, engaging rheumatology, hematology, and infectious disease specialists, is essential for timely diagnosis and management in patients with complex systemic presentations, and other differentials should be considered while awaiting confirmatory testing.

## References

[1] Snyder MJ, Guthrie M, Cagle S (2018). Acute appendicitis: efficient diagnosis and management. Am Fam Physician.

[2] Wong Y, Meehan MT, Burrows SR (2022). Estimating the global burden of Epstein-Barr virus-related cancers. J Cancer Res Clin Oncol.

[3] Münz C (2025). Epstein-Barr virus pathogenesis and emerging control strategies. Nat Rev Microbiol.

[4] Patel PD, Alghareeb R, Hussain A, Maheshwari MV, Khalid N (2022). The association of Epstein-Barr virus with cancer. Cureus.

[5] Thorley-Lawson DA, Hawkins JB, Tracy SI, Shapiro M (2013). The pathogenesis of Epstein-Barr virus persistent infection. Curr Opin Virol.

[6] Henter JI (2025). Hemophagocytic lymphohistiocytosis. N Engl J Med.

[7] Dai X, Fan Y, Zhao X (2025). Systemic lupus erythematosus: updated insights on the pathogenesis, diagnosis, prevention and therapeutics. Signal Transduct Target Ther.

[8] Ranjan S, Kumar S, Nayak H, Panda AK (2025). Epstein-Barr virus infection and its association with systemic lupus erythematosus: systematic review and meta-analysis. Lupus.

[9] Kernder A, Richter JG, Fischer-Betz R (2021). Delayed diagnosis adversely affects outcome in systemic lupus erythematosus: cross sectional analysis of the LuLa cohort. Lupus.

[10] de Almeida Leite RM, Seo DJ, Gomez-Eslava B (2022). Nonoperative vs operative management of uncomplicated acute appendicitis: a systematic review and meta-analysis. JAMA Surg.

[11] Mason RJ, Moazzez A, Sohn H, Katkhouda N (2012). Meta-analysis of randomized trials comparing antibiotic therapy with appendectomy for acute uncomplicated (no abscess or phlegmon) appendicitis. Surg Infect (Larchmt).

[12] Jog NR, James JA (2020). Epstein Barr virus and autoimmune responses in systemic lupus erythematosus. Front Immunol.

[13] Ameer MA, Tariq MA, Zain S, Kabir A, Khawaja M (2024). A rare cause of pancytopenia in systemic lupus erythematosus (SLE) in a young patient. Cureus.

[14] Setiadi A, Zoref-Lorenz A, Lee CY, Jordan MB, Chen LYC (2022). Malignancy-associated haemophagocytic lymphohistiocytosis. Lancet Haematol.

[15] Paydas S, Koçak R, Zorludemir S, Baslamisli F (1997). Bone marrow necrosis in antiphospholipid syndrome. J Clin Pathol.

[16] Bashal F (2013). Hematological disorders in patients with systemic lupus erythematosus. Open Rheumatol J.

[17] Aringer M, Costenbader K, Daikh D (2019). 2019 European League Against Rheumatism/American College of Rheumatology classification criteria for systemic lupus erythematosus. Arthritis Rheumatol.

[18] La Rosée P, La Rosée F (2024). HLH: diagnostics revisited and improved. Blood.

[19] Padalko EY, Bossuyt X (2001). Anti-dsDNA antibodies associated with acute EBV infection in Sjögren's syndrome. Ann Rheum Dis.

